# Special Issue: MAPK Signaling Cascades in Human Health and Diseases

**DOI:** 10.3390/ijms252011226

**Published:** 2024-10-18

**Authors:** Rony Seger

**Affiliations:** Department of Immunology and Regenerative Biology, The Weizmann Institute of Science, Rehovot 7610001, Israel; rony.seger@weizmann.ac.il; Tel.: +972-89343602

## 1. Introduction

In order to survive and fulfil their functions, cells of any organism need to be able to respond to a large number of extracellular factors, also termed extracellular stimuli. These responses are mediated through various regulatory proteins, including intracellular signaling proteins and transcription factors, which eventually lead to the modulation of cellular processes and ultimately determine cell fate. The extracellular stimuli include hormones, growth factors, and cytokines, as well as environmental changes (e.g., stress, temperature change). Most extracellular agents cannot cross the plasma membrane and transmit their signal via membranal receptors, which further activate several intracellular signaling pathways to lead the signals to regulatory molecules either in the cytoplasm or in the nucleus. These intracellular pathways often function through protein kinases that sequentially phosphorylate and activate each other within signaling cascades. The most notable of these are the mitogen-activated protein kinase (MAPK) cascades that are involved in the regulation of essentially all stimulated processes, including proliferation, differentiation, motility, stress response, survival, and apoptosis (for a review see: [1,2,3]. Being such central signaling components, the dysregulation of the MAPK cascades often generates pathologies such as cancer, autoimmune diseases, neurological disorders, and others [4,5,6,7]. In this Special Issue, we cover advances in the study of MAPKs, covering important issues in the regulation and unction of MAPK cascades. These articles should pave the way to a better understanding of stimulated cellular signaling in health and disease.

## 2. The Composition of MAPK Cascades

**Figure 1:**
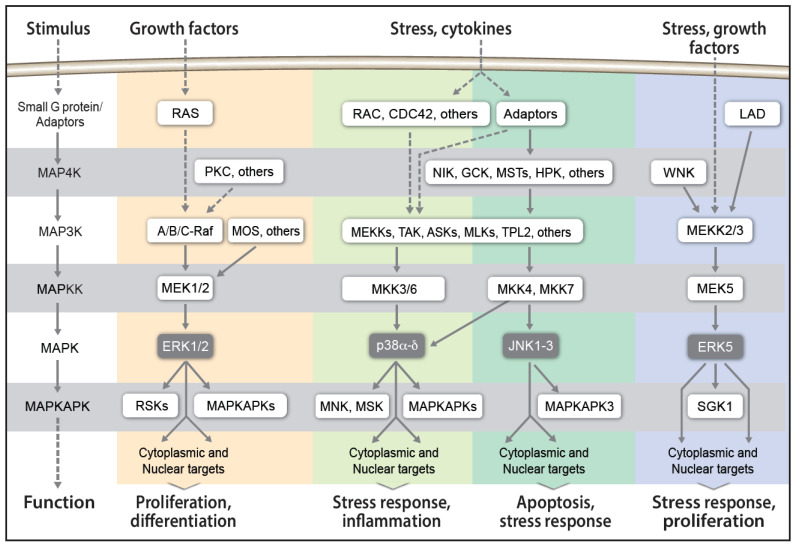
Schematic representation of the four MAPK cascades, without inactivating components. The cascades are named according to the component in the MAPK tier (dark gray). The components of each cascade appear under a different color: ERK1/2—peach, P38α-δ—light green, JNK1-3—darker green, ERK5—lavender. More details are provided in the text.

MAPK cascades are groups of protein serine/threonine kinases (Figure 1) that sequentially phosphorylate and activate each other. They are composed of three to five tiers, depending on the conditions and cell types, where the core cascade is composed of three (MAP3K, MAPKK, and MAPK) and often also upstream (MAP4K) and downstream (MAPKAPK) tiers. The cascades are evolutionarily conserved and have been well-studied in yeasts [8], insects [9,10], and plants [11], which have each three distinct cascades, and in mammals, which have four cascades [12]. The four cascades in mammals that are named according to the components of their MAPK tiers are as follows: (i) extracellular signal-regulated kinase 1/2 (ERK1/2, MAPK1/3 ) [13,14,15], which is composed in most cases of Raf-MEK-ERK-RSK; (ii) p38MAPKα–δ (p38α-δ, MAPK14/11/12/13) [16,17,18], composed mainly of MAP4Ks-MAP3Ks-MKK3/6-p38-MAPKAPKs; (iii) c-Jun-*N*-terminal kinase 1–3 (JNK1-3, MAPK8/9/10) [19,20,21], composed mainly of MAP4Ks-MAP3Ks-MKK4/7-JNK-MAPKAPK3; and (iv) ERK5 (BMK1, MAPK7) [22,23], composed mainly of MEKK2/3-MEK5-ERK5. Many of the components express several alternatively spliced isoforms that often extend the signaling output of the cascades [24]. While MAPK-like proteins such as ERK3/4, ERK7/8 exist as well, they are not considered genuine MAPKs because they do not operate within kinases cascades and because of their different modes of regulation [25]. All these cascades respond to a variety of extracellular signals, but each has a preferential set of activating compounds and regulated processes. Thus, the ERK1/2 cascade preferentially regulates proliferation, differentiation, and migration; p38 regulates mainly stress and immune responses; JNK preferentially regulates stress response and apoptosis; and ERK5 is mainly involved in survival proliferation and sometime also stress responses. In pathologies, ERK1/2 play an important role in cancer, p38 in inflammation, JNK in neurodegenerative diseases, and ERK5 in cancer [4]. All the seemingly linear cascades use similar mechanisms to transmit their signals, but the output of each one may vary according to distinct stimuli [26]. 

## 3. Recent Notable Advances in the Field of MAPK Signaling

Although much information on the activation, regulation and role in diseases of MAPKs have been accumulated over the past three decades, there are still some open questions that need to be addressed in order to understand the regulation of cell fates upon in varying conditions. Many of the pressing questions have to do with the role of MAPK signaling components in diseases and their use as therapeutic targets. In this area, the involvement of ERK1/2 in various cancers has attracted much attention in the past few years. Notably, the full influence of oncogenic Ras on both ERK1/2-dependent transcriptome and phosphoproteome has recently been elucidated [27,28], showing a critical role of ERK1/2 in driving the tumor growth of KRas-transformed cells, acting via a large number of molecular mechanisms. Being such central mediators of various diseases, much effort has been devoted to developing the inhibitors of the components of the cascades. Indeed, efficient inhibitors of components of the four cascades have been developed over the years [29,30,31,32]. Regarding their use in cancer, the best studied inhibitors are those of the ERK1/2 cascade. However, although initially efficient, all of them develop resistance within several months [33]. Therefore, efforts to develop drugs that do not generate resistance are ongoing. This often involves the inhibition of other resistance-inducing pathways such as the inhibition of the androgen receptor in melanoma [34]. In addition, understanding the molecular basis of the inhibitor’s action (e.g., [35]) may lead to the improvement of the drug’s efficacy.

Aside from the role of the MAPK cascades in diseases, some open questions that have been recently addressed include the activation of MAPKs by GPCRs, as well as the mechanisms of regulation of MAPK-induced processes. Although MAPKs’ activation via growth factors is well understood [4], less is known of its activation by GPCRs. A recent study demonstrated that G_αi2_ may influence TCR signaling by sequestering RASA2, thereby promoting RAS activation and stimulating ERK and PI3K to induce proliferation [36]. Another mechanism by which GqPCR induces JNK activation and apoptosis has been researched by our group in the past few years [37,38,39] and is discussed in a review in this Special Issue [3]. Another subject that is still not fully elucidated is the nuclear functions of MAPKs [40,41], particularly the role of their direct DNA binding [42,43]. Interestingly, it was recently shown that ERK2 binds to the cMyc promotor and anchors CDK9 to this region in a kinase activity-independent manner, thus regulating cMyc expression [44]. More studies are required to uncover how general this effect is, and whether it can regulate other MAPK-induced transcriptions as well. Finally, although they have been studied for many years [14], the mechanisms that are involved in specificity determination of MAPKs are still not fully elucidated. Recent important studies in this area have generated information on the HPIP scaffold [45]. In addition, it was shown that distinct scaffolds can couple to allow crosstalk between the signaling by distinct stimuli [46], and structural studies have deciphered the specificity determination of the MKK6-p38 interaction [47]. It is likely that more future studies in this direction will shed more light on this important issue. More state-of-the-art studies on both basic and clinical aspects of the MAPK cascades appear in this Special Issue and are outlined as follows. 

## 4. Recent Advances on the Role of MAPK in Diseases That Are Discussed in This Special Issue

The dysregulation of the MAPK cascades is involved in the induction and progression of various diseases, particularly cancer. Three papers in this issue focused on the involvement of MAPKs in cancer therapy. One of these is a paper by Chen and Park [48], which describes the development of resistance to BRaf and MEK1/2 inhibitors. It is well-known that the activating mutants of the components of the ERK1/2 cascade serve as oncogenes in a high number of cancers [49]. Indeed, the cascade is used as a therapeutic target in various cancers, including melanoma, thyroid cancer, and others, and inhibitors of Raf and MEK are already in use in clinical practice. However, the drugs are not effective in all patients, and even when they are initially beneficial, all patients develop drug resistance within several months. The molecular mechanisms involved in the insensitivity or acquired resistance to these drugs have been studied over the past few years and have been found to include a large number of ERK1/2-related processes [50]. In many cases, there is an elevated expression/activation of activated signaling proteins downstream of the blockade, which leads to a higher ERK activity. Finally, bypassing the blockade can be achieved through the activation of other proliferation-related mechanisms, which is often mediated by a drug-induced reduction of an ERK-dependent negative feedback loop, resulting in the hyperactivation of the upstream components that further activate other pathways (e.g., AKT). The review by Chen and Park offers an update on the mechanisms and steps that are taken to overcome the current challenges when using Raf-MEK inhibitors in clinical practice. 

In the past few years, there has been significant interest in immunotherapy as a tool to combat cancer. Although MAPK cascades are not considered central in the response of cells to immunotherapy, they do participate in some of the processes involved. Some of these processes include the activation of nuclear receptors that are usually regulated by various oncogenes and the activated MAPKs. In two very comprehensive complementary reviews, Dr. Burgermeister describes the crosstalk between MAPK cascades, nuclear receptors, and immunotherapy [51,52], entertaining the idea that a combination of immunotherapy and MAPK inhibition may be beneficial in the cure of various cancers. Nuclear receptors are a group of 48 ligand-activated transcription factors that are divided into several subclasses, including the “classical” endocrine hormones receptors but also other “exploratory” receptors that include immunological mediators which participate in the regulation of processes such as tissue injury and inflammation. Some of these nuclear receptors (mainly metabolic and xenobiotic receptors) may regulate checkpoint protein expression, leading to modification in innate and adaptive immunity and consequently also immunotherapy. Several exploratory nuclear receptors that modulate host immunity are shown in Table 1 of Ref. [51], in which a group of five nuclear receptors are shown to be immune activators and five others as immune suppressors. An example for the first group is HNF4a, whose activity correlates with altered immune cell infiltration and checkpoint gene expression in patients. It was also shown that this nuclear receptor is regulated by ERK1/2, revealing the role of MAPKs in elevating immune response. An example of the second group might be Liver X Receptor (LXR) that acts on the crossroad of metabolism and inflammation to inhibit immune response. This protein was shown to be regulated by the MAPKs JNK and p38, leading to a reduced immune response. In the second review [52], Dr. Burgermeister described the action and crosstalk of classical hormone receptors, again showing that some of them (e.g., FXR, VDR, and RXR) serve as immune activators, while others (e.g., ER, AR, and PR) serve as immune suppressors (Table 1). An example from the first group is that the Retinoid X Receptor (RXR) has been shown to modulate the immune microenvironment and expand tissue-resident macrophages and, thus, progression of cancer. As an example of the suppressive effects, the estrogen receptor has been shown to increase the intracellular PD1 protein and promote immunosuppression mediated by CD4+FOXP3+ Tregs. As mentioned above regarding the exploratory receptors, all the components discussed have been shown to be regulated by or to regulate MAPKs. Therefore, these processes and their regulation may serve as an interaction node between the MAPKs and immunotherapy. More studies are required in order to fully understand this important crosstalk and to uncover a way to utilize it in a cure for cancer. 

Another article with clinical implications that appears in this Special Issue is concerned with the strategies used by enteric bacterial pathogens to modulate MAPKs and host responses. Infectious pathogens, such as *Escherichia coli*, *Shigella*, *Salmonella*, and others, can cause severe diarrhea upon ingestion. Although significant effort has been invested in the search for an efficient drug (aside from antibiotics) to block this effect, the molecular mechanisms involved in these processes are still not fully elucidated. In this Issue, Nandi and Aroeti describe the MAPK cascades as central mediators of the cellular effects of enteric bacteria [53]. They describe the upstream components, such as pattern recognition receptors on the cell surface, that sense bacteria and the way that the signals are transmitted from the receptor to the MAPK cascades using small GTPases (e.g., Ras and Rac), as well as kinase mediators such as TAK1 and Raf that further transmit a signal to the three MAPK cascades, ERK1/2, JNK, and p38. The comprehensive understanding of these mechanisms is important for the development of novel anti-microbial treatments, including the use of clinical MAPK inhibitors, which is a timely endeavor in view of the developing resistance of all used antibiotics. Moreover, the review certainly sheds a new light on receptor-independent intracellular signaling, making the bacterial pathogen–MAPK interface a leading paradigm in developing inhibitors for other diseases as well.

## 5. Recent Advances in MAPK Activity and Effects That Are Discussed in This Special Issue 

The role of MAPKs in distinct cellular processes is covered in one research paper and two reviews in this Issue. One of the research papers by the group of Prof. Engelberg investigated the role of MAPKs, particularly p38α, in various tissues, including several types of skeletal muscles [54]. p38α has been demonstrated in the past as a central player in the regulation of skeletal muscle aging. However, the mechanism, the type of skeletal muscle, and the differences from other tissues were not clear. In the article, the authors followed the levels of MAPKs expression and phosphorylation in several organs as well as several muscle types at varying time points over a period of twenty-four months. They found that in most tissues and conditions, there were no changes in MAPKs’ phosphorylation between the samples, including at the late time points. However, in lungs and in the subtype of skeletal muscle quadriceps, p38α, and to a certain extent also JNK and ERK, shows significantly elevated phosphorylation in aged mice. Interestingly, cell cycle inhibitors, as well as senescence-associated proteins, which are considered to be aging markers, were not elevated in any of the conditions used, thus questioning their generality. The group concluded that MAPK activation in aging is tissue- and muscle type-specific, supporting the notion that the process of aging may be generated in other ways in distinct tissues. 

Insulin-like growth factor 1 (IGF1) is a peptide that affects cells via its unique tyrosin kinase receptor. It demonstrates a ubiquitous expression pattern and regulates the growth of a large number of cells in various tissues. Interestingly, the signaling by IGF1 is somewhat different from that of other growth factors and is heavily involved in the survival of various cells and in cancers. These differences may be related to the nuclear translocation of the IGF1 receptor, which functions as an independent transcription factor. In this Special Issue, Prof. Werner reviewed the various functions of IGF1, its role in activating MAPKs, and the use of its signaling as a molecular target in oncology [55]. The MAPK cascades as well as PI3K/AKT signaling are activated by IGF1 receptor that further interacts with and phosphorylates the adaptor protein IRS1. The phosphorylation of IRS1 on Tyr residues further transmits the signal via adaptor proteins to Ras and PI3K. Aside from this signaling, the IGF1 receptor translocates to the cell nucleus after modification by the ubiquitin-like protein SUMO-1. Interestingly, the nuclear IGF1 receptor colocalizes with ERK1/2 upon stimulation there, extending the signaling and functional repertoire of IGF-1. However, more studies are required in order to fully understand these nuclear effects. Finally, a better understanding of the mechanisms of the IGF1 receptor’s action will largely improve IGF1 receptor-targeted therapies.

The JNK signaling cascade plays an important role in transmitting stress signals and inducing apoptosis. The activation of this cascade by stress is relatively well understood, but much less is known about the activation of the cascade by the G protein-coupled receptor (GPCRs), which may result in stress-independent apoptosis. In this Special Issue, my group reviewed the role of the JNK cascade in the induction of apoptosis through various stimuli and cell lines, in particular in some cells downstream of GqPCRs [3]. Interestingly, we found that in certain cell types, particularly cells of the reproductive system, GqPCRs transmit a signal to JNK by activating a pathway involving PKC-cSrc-MLK3-MKK4/7-JNK. In these cells, the activation of JNK leads to apoptosis, which is required for their functioning. Interestingly, this pathway is heavily regulated by AKT, which phosphorylates and blocks the signaling of MLK3. The stimulation of certain cells results in AKT dephosphorylation and inactivation, a process that alleviates the inhibition of MLK3, and therefore allows the Src signals to proceed, leading to JNK-dependent apoptosis. We also describe a PP2A switch that involves PP2Ac phosphorylation by PKC to inhibit the activity of both PI3K and AKT. Thus, this review provides information on a JNK-induced apoptosis in general and also on GqPCR-induced JNK, which mainly occurs in cells in the reproductive system.

As mentioned above, p38 is composed of four gene products that are translated into four main products (α, β, γ and δ) and at least two other gene products alternatively spliced in p38α [24]. However, all these isoforms seem to have the same consensus phosphorylation site, and therefore, their specificity is not clear. Moreover, the differences between their transient effects in response to stimuli and their chronic effects in diseases are not known. To answer these two questions, Dr. Admon and colleagues used large-scale proteomics and phosphoproteomics analyses to elucidate the differences between p38α and p38β [56]. They found significant differences in the proteome and phosphoproteome between cells expressing active p38α or p38β, which suggests distinct roles for each kinase. Interestingly, constitutive activation induced the adaptation of the cells to chronic activity, so many of the substrates of both p38s were downregulated. Some of the important phosphorylation sites identified were found in p53, Hspb1 (HSP27), and in cytoskeleton-associated proteins. In p53, the main phosphorylation-site (phosphosite) identified was Ser309, which was mainly phosphorylated by p38α of p53-Ser309, suggesting that these p38s may play a role in the induction/regulation of cancer. Overall, this study shows that despite the similarities between p38α and p38β, they are distinct in their substrate recognition both before and after stimulation. More studies are required to fully elucidate the mechanisms that cause these differences.

Overall, this Special Issue provides state-of-the-art information on open questions in the field of MAPK signaling, which is important for understanding these processes, especially the way they affect cell fate, as well as the development and cure of diseases. Finally, this information is important for the development of novel drugs that aim to cure cancer and other diseases.
